# Blindness Caused by Engorgement of the Maxillary Sinus

**Published:** 1858-09

**Authors:** J. Murray

**Affiliations:** Surgeon Dentist, Beaver, Pa


### BLINDNESS CAUSED BY ENGORGEMENT OF THE MAXILLARY SINUS.

(READ BEFORE THE MEDICAL SOCIETY OF BEAVER COUNTY.)

BY J. MURRAY, SURGEON DENTIST, BEAVER, PA.

Mrs. Swager, of Phillipsburgh, Beaver county, Pa., aged thirty-five, sent for me on the 22d day of January. I did not see her till the 23d. I found her in bed much emaciated and apparently rapidly approaching the fatal moment. She had been under medical treatment for five years, but the real cause or seat of the disease had never been suspected. Upon examination, I came to the conclusion very soon, that her disease was engorgement of the antrum. The nasal opening had been closed for the last six months, as was indicated by the dryness of the nostrils. The most acute pain in the face and head, prevented rest or sleep, to any extent. At first sight, every thing seemed unfavorable to a happy termination; the general health was much impaired, the neighboring parts had all become implicated. The bones of the nose had become seriously affected, several exfoliations having already passed off through the palate and nasal fossæ, the largest of which I obtained from the patient for the purpose of exhibiting to this society. When we add to all these unfavorable symptoms, the fact that the patient was blind, and had been so since the 25th of December, 1856; since which time she had never been able to see a ray of light from any luminary, however brilliant, you have the case fully before you in its true and unvarnished color. We looked upon the *age* of the patient as the only circumstance furnishing the least ray of hope for a successful operation. The eye was free of cataract, or opacity of the crystalline lens or its capsule. There was no inflammation, or even weakness, except that arising out of the closing of the lachrymal ducts. The whole appearance was that of *gutta serena* or *amaurosis*.

On examining her mouth, I found, although the teeth were comparatively free from decay, except two roots, that they were nevertheless, coated with tartar. Her gums were inflamed and tumified, and the alveoli were so much wasted, that the roots were left bare, and the teeth considerably loosened.

From this fearful array of evidence the conclusion forced itself upon me that the patient was suffering from engorgement of the antrum, brought on by dental irritation ; that the sinus was filled to the utmost of its capacity, and that it was the mechanical pressure upon the floor of the orbit, destroying the healthy functions of the optic nerve, or in some other way diverting the proper angle of vision, that was causing the blindness.

I concluded to take a few hours to study the case, and make further examination. I appointed the next day at ten o'clock to perform the operation. On my way to the place I called at the office of Dr. J. H. Dickson, and stated the case to him, and requested him to accompany me, but as professional duty called him in another direction, he could not go.

My first object was to ascertain which side of the antrum was affected. Having decided that it was the right, I extracted the first and second molars on that side, and from the socket of the second molar I pierced the antrum with a spear shaped trocar. I then enlarged the aperture to about the size of a small quill. The extreme debility and nervousness of the patient prevented me from making the opening as large as I could have desired. From this opening considerable pus mixed with blood escaped. I then injected the cavity four or five times with diluted tinct. of myrrh. I also extracted a molar on the left side, that I suspected to be the cause of some irritation. The patient was then put to bed and I left her. I saw her the next day, she was decidedly better. The nasal openings were re-established, and the discharge from the nostrils abundant. She had not had a paroxysm of pain in her head since the operation. She had slept comfortably, and had an improved appetite.

I then advised her husband to call in a physician in whom he had confidence, as his wife would need some constitutional treatment. Dr. J. H. Dickson was called in on the 26th of January. I continued to inject the cavity with diluted tinct. of myrrh several times every day for a few days, intending to use something more stimulating. But the patient improved so rapidly that I concluded not to resort to the solution of nitrate of silver.

There was no improvement in the sight until the night of the 29th of January, six days after the operation. That night she thought she could see a ray of light on the right side of the nose. She requested her husband to bring the candle near her, and on passing her hand between the candle and her eyes, she could discern the opacity of the hand. Her sight improved so much through the night, that the next morning she could see the posts of the bedstead. The next day she could walk through the room, avoiding chairs and other obstructions.

On the third of February, her general health was so much improved that she could be out of bed half the day, had an improved appetite, and could now see the color of her child's hair and eyes, at which she expressed great gratification. She could also discern the small checks in her child's apron.

At this visit I extracted another root and cleansed all the remaining teeth in the superior maxillary of their salivary calculus. There are still some loose teeth that *ought* to be extracted if the consent of the patient could be obtained.

April 14. At this date I am able to report Mrs. Swager as enjoying as good health as she ever did in any former part of her life; able to take charge of her house and family, with sufficient sight to attend to all the ordinary business of her family. Her sight is not yet perfect, but improving every day, and from what improvement has already taken place in so short a time, there is good ground to hope that she will eventually be fully restored.

She has never yet submitted to the extraction of the remaining bad roots.

